# In search of environmental risk factors for obsessive-compulsive disorder: Study protocol for the OCDTWIN project

**DOI:** 10.21203/rs.3.rs-2897566/v1

**Published:** 2023-05-11

**Authors:** David Mataix-Cols, Lorena Fernández de la Cruz, Elles de Schipper, Ralf Kuja-Halkola, Cynthia M. Bulik, James J Crowley, Janina Neufeld, Christian Rück, Kristiina Tammimies, Paul Lichtenstein, Sven Bölte, Jan C. Beucke

**Affiliations:** Karolinska Institutet; Karolinska Institutet; Karolinska Institutet; Karolinska Institutet; University of North Carolina at Chapel Hill; University of North Carolina at Chapel Hill; Karolinska Institutet; Karolinska Institutet; Karolinska Institutet; Karolinska Institutet; Karolinska Institutet; MSH Medical School Hamburg

**Keywords:** obsessive-compulsive disorder, monozygotic twins, discordant twins, environmental risk factors, prevention, early intervention

## Abstract

**Background:**

The causes of obsessive-compulsive disorder (OCD) remain unknown. Gene-searching efforts are well underway, but the identification of environmental risk factors is at least as important and should be a priority because some of them may be amenable to prevention or early intervention strategies. Genetically informative studies, particularly those employing the discordant monozygotic (MZ) twin design, are ideally suited to study environmental risk factors. This protocol paper describes the study rationale, aims, and methods of OCDTWIN, an open cohort of MZ twin pairs who are discordant for the diagnosis of OCD.

**Methods:**

OCDTWIN has two broad aims. In Aim 1, we are recruiting MZ twin pairs from across Sweden, conducting thorough clinical assessments, and building a biobank of biological specimens, including blood, saliva, urine, stool, hair, nails, and multimodal brain imaging. A wealth of early life exposures (e.g., perinatal variables, health-related information, psychosocial stressors) are available through linkage with the nationwide registers and the Swedish Twin Registry. Blood spots stored in the Swedish phenylketonuria (PKU) biobank will be available to extract DNA, proteins, and metabolites, providing an invaluable source of biomaterial taken at birth. In Aim 2, we will perform within-pair comparisons of discordant MZ twins, which will allow us to isolate unique environmental risk factors that are in the causal pathway to OCD, while strictly controlling for genetic and early shared environmental influences. To date (May 2023), 43 pairs of twins (21 discordant for OCD) have been recruited.

**Discussion:**

OCDTWIN hopes to generate unique insights into environmental risk factors that are in the causal pathway to OCD, some of which have the potential of being actionable targets.

## BACKGROUND

Despite dedicated research and some breakthroughs in the scientific understanding of relevant neurobiological and psychosocial factors, the causes of obsessive-compulsive disorder (OCD) remain largely unknown. Efforts to identify associated genetic variants are well underway through unprecedented international collaboration [1-3]. The identification of specific environmental factors that confer risk, and may interact with genetic factors, is at least as important as identifying genetic variants [4]. This could be regarded as a priority because some environmental risk factors may be amenable to prevention or early intervention strategies [5]. Unfortunately, little progress has been made in this area, primarily because the identification of environmental risk factors that operate independently from genetic factors is challenging.

Genetically informative studies, specifically those employing the discordant monozygotic (MZ) twin design, are ideally suited to test whether the association between an environmental measure and an observed phenotype (e.g., OCD) is likely to be consistent with a causal effect since they provide strict control of both genetic and early shared environmental effects. In Sweden, we have begun an ambitious project to create the world’s first cohort of MZ twin pairs who are discordant for a diagnosis of OCD. In this protocol paper, we describe the study rationale, aims, and methods of the OCDTWIN project.

**Figure 1:**
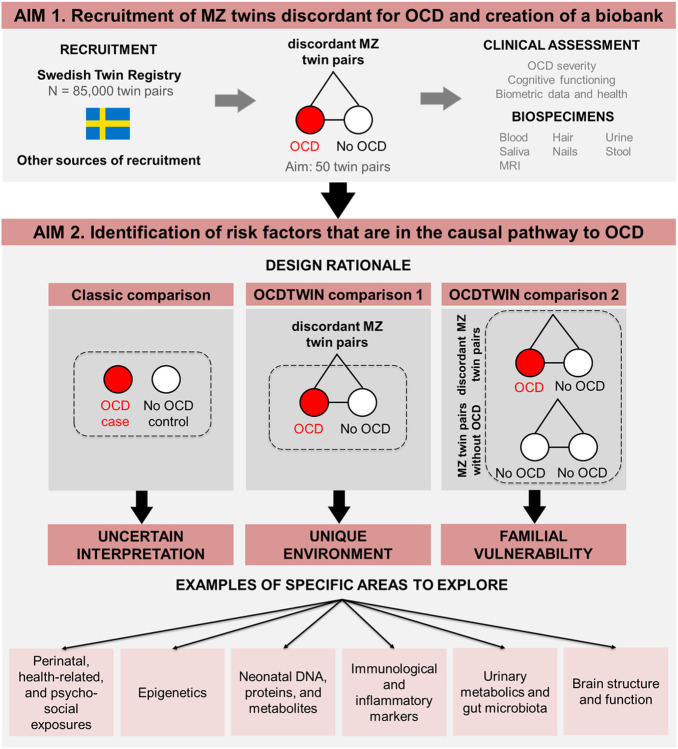
Study aims and design rationale. ODCTWIN primarily aims to recruit a cohort of MZ twins who are discordant for OCD (AIM 1) and identify environmental risk factors that are in the causal pathway to OCD (AIM 2). The results of traditional case-control designs are difficult to interpret because they are unable to effectively control for familial confounders (AIM 2, classic comparison). MZ twins discordant for OCD provide a unique opportunity to isolate environmental risk factors that are unique to each individual, while controlling for measured and unmeasured confounders, including shared genetic factors, and early life environmental effects (AIM 2, comparison 1). Even though our main interest is to identify unique environmental effects, we will also compare affected twin pairs (where at least one twin has OCD) with unaffected twin pairs to identify effects that can be attributed, at least in part, to familial vulnerability (AIM 2, comparison 2).

The broad study aims are two-fold (for a visual summary, see Fig. 1). In *Aim 1*, we are recruiting MZ twin pairs from across Sweden, conducting thorough clinical assessments, and building a biobank of specimens, including blood, saliva, urine, stool, hair, nails, and multimodal brain imaging. A wealth of information regarding early life exposures (e.g., perinatal variables, health-related information, psychosocial stressors) is available through linkage with the nationwide registers [6] and the Swedish Twin Registry [7, 8]. Blood spots stored in the Swedish phenylketonuria (PKU) biobank are available to extract DNA, proteins, and metabolites, providing an invaluable source of biomaterial taken at birth [9]. In *Aim 2*, we will identify variables that are in the causal pathway to OCD through within-pair comparisons of discordant MZ twins. This will allow us to isolate unique environmental factors while strictly controlling for genetic and shared environmental influences. The ultimate goal is to generate new insights into the potentially modifiable causes of OCD.

## METHODS

### Aim 1. Recruitment of MZ twins discordant for OCD and creation of a biobank

#### Participants and recruitment sources

OCDTWIN aims to recruit a minimum of 50 MZ twin pairs (n = 100 unique individuals) discordant for OCD aged 16 years and older (see Power Considerations section below). Control twin pairs without a diagnosis of OCD are available from the ongoing Roots of Autism and Attention-Deficit/Hyperactivity Disorder (ADHD) Twin Study in Sweden (RATSS) project [10], which uses largely identical procedures. Finally, we will also recruit twin MZ pairs concordant for OCD diagnosis. By recruiting both discordant and concordant twin pairs, we will be able to appropriately represent the source population with regards to exposure variables and covariate distributions, and account for this in statistical analyses, both in within-pair analyses and standard (between individual) analyses (see General statistical framework for details).

The twins are recruited from different sources. The main source of recruitment is the Swedish Twin Registry, the largest and most comprehensive twin register in the world [7, 11]. All the cohorts listed in Table 1 contain validated measures of obsessive-compulsive symptoms, allowing identification of MZ pairs potentially concordant or discordant for these symptoms. Furthermore, the Swedish Twin Registry has been linked with the National Patient Register [12], allowing for the identification of twins who have been diagnosed with OCD in specialist services across Sweden. Crucially, participants in the Swedish Twin Registry have provided informed consent to be contacted for research purposes. Other sources of recruitment are the Swedish OCD interest organization (*Svenska OCD-förbundet*) and media advertisements.

#### Procedures

Potential participants identified via any of the sources described above receive a study invitation letter including information about the OCDTWIN project via regular mail. Interested individuals contact the research team. This is followed by a screening phone call to assess eligibility for participation.

Inclusion criteria are: MZ twins, at least one member of the twin pair has a lifetime diagnosis of OCD, both twins consent to participate, are literate in Swedish, and are willing to travel to Stockholm for the assessment. Twins who want to participate in the study but do not wish to travel can still participate in the clinical assessments via telephone and send a subset of biological samples in the post. Information on zygosity is available from the Swedish Twin Registry, but is confirmed by genotyping of saliva or whole-blood derived DNA using a whole-genome covering SNP array [13].

Exclusion criteria include: organic brain disorder, brain injury, epilepsy or an acute mental disorder that may interfere with the evaluation (e.g., psychosis, bipolar disorder). Additional exclusion criteria for the Magnetic Resonance Imaging (MRI) scans include: previous brain surgery, metal implants or medical devices containing metal (e.g., pacemaker), claustrophobia, pregnancy, morbid obesity, or large tattoos. Twins not currently eligible for the MRI scan can still participate in the remaining assessments and can have the scan at a later stage (e.g., in the case of pregnancy).

Twin pairs meeting inclusion criteria are mailed a questionnaire package and invited for a full testing day in Stockholm. Data acquisition consists of a full day of evaluations, including a detailed clinical interview, neurocognitive testing, physical examination, collection of biological samples, and an MRI scan. Table 2 summarizes the collected information and the instruments used. All biological specimens are deposited at the Karolinska Institutet biobank, according to standard protocols.

Participants are reimbursed for their lost working hours. Additionally, participants receive a gift card worth SEK 500 (~ 45 €) for each of the three following parts of the study: (1) clinical assessment, (2) biological samples, and (3) MRI scan (i.e., SEK 1500 [~ 135 €] in total). For twins hailing from outside Stockholm, ground transportation or airfares and accommodation are provided.

### Aim 2. Identification of risk factors that are in the causal pathway to OCD

#### Design rationale

Genetically-informative studies, in particular those employing the discordant MZ twin design, are ideally suited to test whether the association between an environmental measure (e.g., medical complications at birth) and an observed phenotype (e.g., OCD) is likely to be consistent with a causal effect because they provide excellent control of many potential confounders, including genetic factors and shared environmental influences. Because MZ twins are genetically identical, and grow up largely in the same environment, any observed phenotypic differences between members of a MZ twin pair (e.g., one twin is affected and the other is not) may be attributable to non-shared environment. In contrast to studies comparing a sample of cases *vs.* controls (classic comparison, Fig. 1), or even relatives of cases, MZ twins discordant for OCD provide a unique opportunity to isolate environmental risk factors that are unique to each individual, while controlling for a myriad of measured and unmeasured confounders, such as genetic factors, sex, age, parental effects, as well as shared *in utero* and early life environmental effects (comparison 1, Fig. 1).

#### General statistical framework

Within-pair differences between affected MZ twins with OCD and their co-twins will be analyzed in a generalized estimating equation (GEE) framework, accounting for dependencies between twins in pairs using cluster-robust standard errors. In what is commonly referred to as co-twin control design, we will examine within-pair associations by analyzing data conditioned on pairs (fixed-effects regression) [14-16]. Results from these analyses are automatically adjusted for any confounding factors that are shared between twins in a pair [17], particularly genetic factors, since MZ twins are genetically identical (comparison 1, Fig. 1). Even though our main interest is to identify unique environmental effects, we will also compute a standard association (that is, individuals with OCD *vs.* controls, regardless of co-twin OCD status) by re-weighting the data by sampling probability and will thus recover the association in the source population, making it possible to identify effects potentially attributable, at least in part, to familial vulnerability (comparison 2, Fig. 1). In addition, all twin pairs, regardless if recruited as concordant or discordant, will contribute to analyses of within-pair associations between other variables of interest than OCD diagnosis, where they may be discordant, such as scores on OCD severity scales.

#### Register-based data

The Swedish national registers contain administrative records from the entire population prospectively collected over several decades [6]. Data from different registers can be linked by using the personal identification number assigned to all Swedish residents at birth or immigration [18]. We will have access to a wide range of early life exposures, such as perinatal and early-life health-related variables, that may have resulted in differentially exposed twins. For twins recruited via the Swedish Twin Registry [7, 8], a wealth of prospectively collected data (parent and twin-reported) on environmental exposures are available for analysis. The Child and Adolescent Twin Study in Sweden (CATSS) cohort of the Swedish Twin Registry, where most participants are recruited from, has been described in detail elsewhere [19]. Importantly, the information from the Swedish Twin Registry can be linked to the above-mentioned national registers. For a list of linked registers and examples of available variables, see Table 3. Because we carefully record the date of OCD symptom onset and diagnosis of the affected twins, we will be able to identify exposures that preceded symptom onset.

#### Epigenetics – methylation analyses

Current neurobiological models of OCD implicate epigenetic mechanisms in the etiology of OCD [20]. However, the literature is limited. Our design is ideally suited for the identification of epigenetic changes, as the genomes of MZ twins are identical, potentially allowing for the observation of changes in the epigenome in absence of genetic variation between twins. DNA methylation analysis, which has been previously studied in neurodevelopmental disorders [21] and in OCD [22], will be used to determine differential methylation in the affected twin sibling, compared to the unaffected co-twin. Genome-wide methylation analysis will be used first, given the limited evidence of methylation changes in OCD. Second, we will follow the general approach of a previous epigenetic study in OCD [22], which specifically examined DNA methylation profiles of selected loci that had been associated with OCD in previous genome-wide association studies (GWAS) [23-25]. However, previous GWAS of OCD were severely underpowered. Our proposed analyses are timely as the largest GWAS study conducted to date, including approximately 45,000 cases and 30 genome-wide-significant loci, is nearing completion. We hypothesize that affected twins will exhibit differential methylation at genes identified by this GWAS, compared to their unaffected co-twins. Analyses of genome-wide methylation and methylation profiles of selected genes will be performed using array-based specific DNA methylation analysis. This array targets > 935,000 CpG sites at single nucleotide resolution, including 99% of RefSeq genes and 96% of CpG islands can be analyzed. Possibly differentially methylated regions will be confirmed by pyrosequencing or nanopore sequencing. As the blood-derived DNA is a mixture of the blood cell type specific methylation patterns, we will collect information about the cell counts as well as correct bioinformatically if there are any putative differences due to cell populations [26].

As it is a priority of OCDTWIN to identify the epigenetic effects of unique environment while controlling genetic effects, several additional genetic mechanisms will be studied in order to confirm identical genomes in affected and unaffected twins. This includes chromosomal mosaicism, post-zygotic mutation, and mutations of mitochondrial DNA [27]. To assess the landscape of genetic variants among the twins both for somatic and germline, we will use whole genome sequencing (WGS) [28] and high-density DNA microarrays. DNA microarrays can be used for detection of large CNVs using multiple analysis programs, and the variations found in samples will be compared to control twins, other available controls, and databases to identify the frequency and functionality of the variants identified. Furthermore, polygenic risk scores can be calculated and incorporated to all analyses within the OCDTWIN project. WGS can identify rare post-zygotic somatic mutations in the twins. Additionally, rare, damaging variants will be investigated for putative liability variants. Identified somatic and selected damaging germline variants will be subject to technological validation by Sanger sequencing or using digital droplet PCR.

#### Neonatal blood spots

The Swedish PKU biobank [9] contains neonatal blood spots from all children born in Sweden since 1975. Participating twins consent to the use of these blood spots to extract DNA, proteins, and metabolites, providing an invaluable source of biomaterial taken at birth. In other disorders, important discoveries have been made using neonatal blood spots. For example, persons who develop psychosis have lower levels of certain acute phase proteins (APPs) at the time of birth [29]. APPs are central to innate immune function as well as central nervous system development. Prior studies [31] have demonstrated a high genotyping call rate using whole genome amplified DNA from Swedish blood spots collected from 1975 to 2002. Two 3 mm punches from the blood spots are incubated in 200 μl 1x phosphate buffered saline for 2 hours at room temperature on a rotary shaker (900 rpm), yielding an eluate of proteins such as acute phase proteins and antibodies as well as other metabolites (e.g., vitamin D, cytokines, etc.). DNA is then extracted (~ 40–150 ng), only a portion of which (10 ng) is whole genome amplified (Repli-g screening kit, Qiagen). The unamplified DNA retains methylation marks and can be used for epigenetic profiling and/or CNV validation. The amplified DNA can be used for array genotyping, exome sequencing or whole genome sequencing. These analyses will be conducted in collaboration with colleagues at the Statens Serum Institut in Copenhagen, Denmark.

#### Immunology/inflammation

Pediatric Autoimmune Neuropsychiatric Disorder Associated with Streptococcal Infection (PANDAS) can be viewed as an example of a gene-environment interaction leading to OCD [32]. In PANDAS, a relatively common infection appears to represent an environmental stressor that can trigger OCD in a few genetically vulnerable cases. In support of this idea, we have recently reported that while *in utero* and early life infections are associated with a subsequent risk of OCD, the associations attenuated to the null in sibling models [33]. This suggests that familial or genetic factors explain the association between these early-life infections and OCD. In other words, infections may only trigger obsessive-compulsive symptoms in genetically vulnerable individuals. Through register linkage, we will be able to test whether affected twins are more likely to have had documented infections in early childhood, compared to their unaffected co-twins, in OCD-discordant pairs. In addition, the following markers will be tested in blood: complete blood count (CBC), erythrocyte sedimentation rate (ESR), CRP, TSH, T4, anti-TPO, ferritin, autoantibodies (e.g., transglutaminase-Abs, ANA, Histone-Abs), creatinine, cystatin-C, ALAT, protein fractions, complements, IL-1-β, IL-6, IL-8, IL-10, and TNF-α. In line with our statistical approach, differences between affected and unaffected members of a twin pair will be attributable to disease-state (e.g., response to a chronic illness), whereas differences between affected pairs and healthy control pairs may be interpreted as being potentially attributable to trait immunological or vulnerability factors.

#### Urinary metabolics and gut microbiota

By comparing urinary metabolics and gut microbiota within and between twin pairs, we aim to explore an additional etiological pathway that has been recently suggested [34]. Using urinary samples, metabolic phenotyping will involve high-resolution proton nuclear magnetic resonance (hydrogen-1 nuclear magnetic resonance; 1H NMR) spectroscopy coupled with mathematical modeling approaches to identify metabolic variation associated with OCD discordance in urine and plasma. Metabolic profiles are measured on a 600 MHz 1H NMR spectrometer using standard one-dimensional NMR experiments optimized for quality, sensitivity, and solvent suppression. Liquid chromatography-mass spectrometry (LCMS) may be applied to extend the metabolic characterization of this sample set. LCMS is a complementary technique to 1H NMR spectroscopy with greater sensitivity and wider metabolome coverage. Using fecal samples, gut microbiota will be investigated, which has emerged as an important functional node within the gut-brain axis [35]. There is increasing interest in the relative potential of the gut microbiota and allied gastrointestinal systems to modulate behavioral functions implicated in psychiatric disorders, including OCD [34]. The determination of gut microbiota will be based on the quantification of evolutionary conserved DNA sequences [36]. In microbes, ribosomal RNA (rRNA) genes are transcribed from the ribosomal operon as 30S rRNA precursor molecules and then cleaved by RNaseIII into 16S, 23S, and 5S rRNA molecules. Because 16S rRNA is the most conserved of these three rRNAs, it is often referred to as the “evolutionary clock” and, following amplification into 16S rDNA, is highly suitable for the identification and classification of the entire microbial community present in an environmental entity, such as the gut. The total microbial population in human fecal samples will be determined using two state-of-the-art methods, namely 16S rDNA pyrosequencing and 16S rDNA sequencing.

#### Brain

Individuals with OCD display subtle difficulties in neuropsychological tasks of motor and cognitive inhibition, performance monitoring, cognitive flexibility, and emotional processing [37]. Consistently, structural and functional neuroimaging studies have found involvement of specific fronto-striato-thalamic and parietal systems in OCD [37], although causal relationships cannot be established. It is unclear whether differences between OCD cases and controls represent pre-existing vulnerabilities that precede the onset of the disorder or are environmentally or behaviorally mediated. In support of the former view, a number of studies have found that individuals with OCD and their unaffected first-degree relatives share similar cognitive and neural features [e.g., 38, 39, 40]. However, as siblings only share about 50% of their genes, it is still unclear whether these findings reflect genetic vulnerability or environmentally-mediated risk factors. In support of the latter view, variables such as living with a chronic illness are suspected to induce neuroplastic changes in the brain of individuals with OCD [41-44]. Similarly, medication may represent another unique environmental event affecting brain structure in OCD, as indicated by recent mega-analyses [45, 46]. The discordant MZ design is ideally suited to understand what brain findings may be secondary to environmental exposures, such as use of medication.

MRI data are acquired on a 3T General Electric 750 MR scanner (equipped with a 32-channel head coil) at the MR Research Center at Karolinska Institutet. T1-weighted images are acquired using a high-resolution BRAVO 3D sequence, using the following parameters: TR/TE = 8.2/3.2; 172 slices; FOV: 240; 256x256; 1x0.94x0.94 mm; flip angle = 12 degrees. Voxel-based morphometry analyses will determine whether gray matter volume differences in cingulate cortex and basal ganglia areas observed in previous meta-analyses [47] can be attributed to unique environmental risk.

Diffusion tensor imaging (DTI) measurements of white matter microstructure are acquired using High AngulaR Diffusion Imaging (HARDI) with 60 directions and 61 slices, Dual spin Echo Epi2ks axial; TR/TE: 8000/99; FOV 96x96; 8 b0 images, b-value: 1000 s/mm^2^. Fractional anisotropy (FA) and white matter volume analyses will help determine whether white matter differences observed in previous meta-analyses [48] and mega-analyses [49] are associated with unique environmental risk factors.

Resting-state functional magnetic resonance imaging involves acquisition of 205 echo-planar images (EPI) using the following parameters: 45 slices; interleaved ascending slice order; TR/TE = 3000/30ms; FOV = 288; 96x96; 3x3x3 mm; flip angle = 90 degrees. Degree connectivity analysis will be performed[50], revealing local and distant “hubs” of connectivity, as well as traditional seed-based whole-brain correlation analysis to determine striatal connectivity [51, 52] and default mode network (DMN) [53].

Spatial associations between within-pair differences in whole-brain measures and whole-brain gene expression patterns will be explored. The Allen Human Brain Atlas [54] will be used to test for associations between brain structure and connectivity differences (results from within-pair comparisons) and gene expression in a previously described manner [55-57] without requiring information from blood or saliva samples, which can however potentially be integrated in subsequent analyses [57]. The spatial similarity between transcriptional profiles of the entire transcriptome atlas and within-pair differences in brain measures observed in our study population will be quantified. Histogram distributions of spatial similarity values will reveal genes where the genetic expression pattern is significantly associated with brain structure and connectivity maps. Moreover, we will particularly focus on neurogenetic processes by investigating specific gene ontology (GO) term analysis for “neuro” annotations, as described previously [55, 56]. Finally, we will focus on specific genes strongly suspected to be associated with OCD (such as DLGAP3 [58-61] or NRXN1 [62]) and also new genes uncovered in the latest GWAS.

#### Power considerations

**Figure 2:**
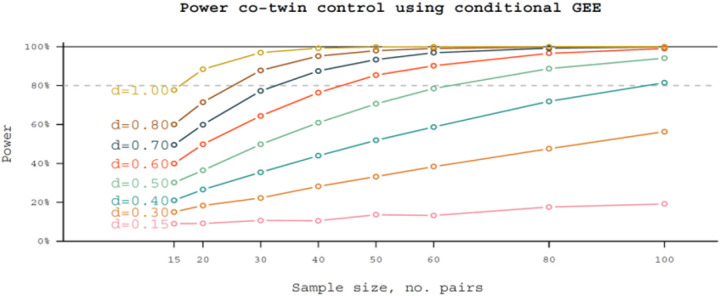
Number of discordant twin pairs needed for power per different effect sizes. *“d”* refers to average difference, measured in standard deviations, between affected and unaffected twins, similar to Cohen’s *d*.

Given the novelty of the approaches presented, power analyses are necessarily tentative. Although we have a variation in distribution of variables, we have performed a power calculation for continuous, normally distributed variables in a co-twin control design using GEE analytic framework (i.e., fixed-effects linear regression) (see Fig. 2). With 50 discordant MZ pairs, we will have approximately 80% power to detect medium to strong associations (Cohen’s *d* of approximately 0.6). Publications emerging from the ongoing RATSS project [63, 64] suggest that the proposed sample sizes can yield meaningful results.

## DISCUSSION

To our knowledge, OCDTWIN represents the world’s only cohort of MZ twins discordant or concordant for OCD. The project hopes to generate unique insights into environmental risk factors that are in the causal pathway to OCD, some of which have the potential of being actionable targets. If successful, this could be a first step towards fulfilling the long-held ambition of preventing the development of OCD or, if this were not possible, intervene as early as possible to prevent the long-term medical [65] and socioeconomic [66, 67] consequences of the disorder.

Some challenges for the success of the project are participant recruitment, uncertainty regarding statistical power for some of the proposed analyses, and interpretation of the results. Initially, we aim to recruit at least 50 discordant pairs of twins. At the time of writing (May 2023), a total of 43 MZ twin pairs (86 individuals) have already been recruited. Twenty-one of those MZ pairs are discordant for OCD diagnosis and 22 are concordant. Control twins are available from the parallel RATSS study [10]. Our main recruitment source, the CATSS cohort within the Swedish Twin Registry [19], is still actively recruiting at a rate of approximately 3,000 new twins per year, providing a sustained source of potential study participants. Data collection will continue for at least the next two years. If we secure additional funds, we aim to continue recruiting participants beyond the planned 50 pairs, thus increasing statistical power. The study is currently limited to Swedish residents and to participants who are 16 years or older. However, we may consider expanding to twin pairs from other countries in the future. There is a risk that some of the younger twins identified as unaffected have not had time to develop OCD by the time of their participation, as the youngest participants may be 16 years old. We plan to follow up the twins in the registers to capture any new diagnoses of OCD after they have been recruited to OCDTWIN. Some of the described methods and analysis plans may be obsolete by the time we are ready for data analysis. We are collecting hair and nails but have no specific plans for analysis at the time of writing. We will closely follow methodological developments in the field.

While the primary aim of OCDTWIN is the identification of environmental risk factors that are in the causal pathway to OCD, we will collect a wide range of exposures from birth (e.g., perinatal complications, birth order, birth weight), childhood (e.g., early infections, bullying and other traumatic experiences), and up to the time of participation in the study (e.g., current medication use). While the interpretation of results regarding early exposures will be relatively straightforward because these exposures will precede the onset of OCD symptoms, the interpretation of results based on more recent exposures will be more challenging. For example, differences between affected and unaffected twins on a given brain measure could be attributable to environmental exposures accumulated during a lifetime, including changes secondary to chronic illness or medication use. Even in this scenario, the results will still be informative because the nature of the design minimizes the influence of genetic and shared environmental factors, and an association could reveal important, potentially actionable mediators. However, the interpretation of the results will differ according to each specific exposure and whether temporal precedence can be clearly established.

There are additional challenges associated with the discordant MZ twin design. Our approach assumes that MZ twins are genetically identical. However, post-zygotic mutations are known to occur and can be specific to one twin in a pair [28], which could explain OCD discordance in some pairs. On the other hand, this will provide a unique opportunity for genetic discovery. Another potential challenge is twin chorionicity, which is often unknown for adult twins. MZ twins can be sub-classified according to whether they shared the same placenta or not. For example, in a schizophrenia study, concordant MZ pairs were estimated to be more likely to have shared a single placenta, whereas discordant MZ pairs appeared more likely to have separate placentas [68]. Whether and how post-zygotic mutations and chorionicity can impact the interpretation of our results is unclear but will be considered.

The project is expected to generate many scientific outputs. All resulting papers will be deposited in preprint repositories (e.g., bioRxiv, PsyArXiv) to ensure immediate access to the scientific community. We will publish the results in specialized peer-reviewed journals that allow open access formats. Through partnership with other researchers who are collecting similar twin data in other disorders in Sweden, it may be possible to establish which findings are specific to OCD or shared with other neuropsychiatric conditions. OCDTWIN will collect nearly the same data as the RATSS study [10], which focuses on autism and ADHD. Similarly, the ongoing CREAT (Comprehensive Risk Evaluation for Anorexia nervosa in Twins) study focuses on MZ twins who are discordant for anorexia nervosa [69]. Both these cohorts will provide additional opportunities for collaboration.

## Figures and Tables

**Table 1:** Cohorts in the Swedish Twin Registry used for recruitment purposes and criteria for potential participant selection.

Twin cohort	Num. of individual twins as of November 2022	Obsessive-compulsive symptom measurement	Criteria for selection for potential inclusion
**CATSS-18** (born 1992-)	15,400	Brief Obsessive-Compulsive Scale (BOCS),^1^ *17 items*	Top 5% scores on the BOCS.
**CATSS-24** (born 1992-)	7,639	Obsessive-Compulsive Inventory – Revised (OCI-R), *12 items (hoarding and neutralizing scales not included)* Self-reported question: *Have you ever been diagnosed with (or suspected that you have) OCD?*	Top 5% scores on the OCI-R or answered yes to the self-reported question.
**DOGSS** (born 1993–1995)	450	Kiddie - Schedule for Affective Disorders and Schizophrenia for school-aged children, Present and Lifetime Version (K-SADS-PL),^3^ *obsessive-compulsive disorder categorical diagnosis*	Met diagnostic criteria for obsessive-compulsive disorder on the K-SADS-PL.
**YATSS** (born 1985–1992)	6,870	OCI-R, *12 items (hoarding and neutralizing scales not included)*	Top 5% scores on the OCI-R.
**STAGE** (born 1959–1985)	43,000	7-item obsessive-compulsive disorder questionnaire Self-reported question: *Have you ever been diagnosed as having an obsessive compulsive disorder?* (yes/no)	Top 5% scores on the 7-item obsessive-compulsive disorder questionnaire or answered yes to the self-reported question.

Abbreviations: CATSS, *Child and Adolescent Twin Study in Sweden; DOGSS, Developmental Outcomes in a Genetic Twin Study in Sweden; YATSS, Young Adult Twins in Sweden Study; STAGE, Screening Twin Adults: Genes and Environment study*.

**Table 2:** Study variables and collection.

STUDY VARIABLE	MEASURED BY
**Socio-demographic and clinical information**	
Demographics	Self-reported questionnaire
Zygosity	Self-reported questionnaire, confirmed by genotyping of saliva or whole-blood derived DNA using a whole-genome covering SNP array
Treatment history (medication, therapy)	Clinician-administered interview
**Psychiatric disorders**	
Structured Clinical Interview for DSM-5 (SCID-5)	Clinician-administered interview
**OCD severity and symptom dimensions**	
Yale-Brown Obsessive Compulsive Scale (Y-BOCS), Symptom Severity and Checklist	Clinician-administered interview
Obsessive Compulsive Inventory-Revised (OCI-R)	Self-reported questionnaire
**Cognitive function**	
Similarities, Vocabulary, Information, Block design, Matrix reasoning, and Visual puzzles from the Wechsler Adult Intelligence Scale-fourth edition (WAIS-IV)	Clinician-administered test
Reading the mind in the eyes (RTMITE)	
Fragmented Picture Test (FPT)	
Edinburgh Inventory modified (Handedness)	Self-reported questionnaire
**Biometric data and general health**	
Height	Physical exam by nurse
Weight	
BMI	
Head circumference	
Blood pressure	
Pulse	
Medical and Clinical Genetic Questionnaire	Self-reported questionnaire
**Biospecimens**	
Blood	Collected by nurse
Saliva	
Hair	
Nails	
Urine	Collected by participant
Stool	
**Brain imaging**	
High resolution structural scan (T1)	Study coordinator
Diffusion Tensor Imaging (DTI)	
Resting state functional Magnetic Resonance Imaging (rsfMRI)	
**Other measures**	
Adult Self Report (ASR)	Self-reported questionnaire
Adult Autism Spectrum Quotient (AQ)	
Sensory Profile Adolescent/Adult (SP-A)	
Swedish Eating Assessment for Autism Spectrum Disorders (SWEAA)	
Attention-Deficit/Hyperactivity Disorder Self Report Scale (ASRS)	
Camouflaging Autistic Traits Questionnaire (CAT-Q)	

**Table 3:** Registers and examples of variables available from each participant.

Register name	Examples of available variables
**Swedish national registers**	
National Patient Register [12]	Information on all inpatient and outpatient contacts at all hospitals and specialist centers. Includes primary and supplementary diagnoses based on the International Classification of Diseases codes, including somatic (e.g., autoimmune diseases, infections, allergies, respiratory diseases) and psychiatric disorders (e.g., trauma and stress related disorders, mood disorders, substance use disorders).
Medical Birth Register [70]	Information on antenatal, obstetric, and neonatal care, including mother’s parity, mother’s age, type of delivery, obstetric complications (e.g., preeclampsia, gestational diabetes, antepartum or postpartum hemorrhage), gestational age, birth weight, birth order, APGAR scores, neonatal hypoglycemia, neonatal jaundice, neonatal infections, neonatal respiratory distress, congenital malformations, etc.
Prescription Drug Register [71]	Individual-level data for all prescriptions dispensed for in- and outpatients, including type of medication registered using Anatomical Therapeutic Chemical (ATC) Classification System codes, dosage, prescription date, prescriber, pharmacy, etc.
**Swedish Twin Registry**	
	Variables collected via questionnaires in different waves, including somatic and mental health, personality development, vaccinations, substance use, physical activity or psychosocial adaptation and environment (e.g., traumatic events, school problems, friendships, bullying victimization/perpetration).

## Abbreviations

3T: 3 Tesla
ADHD: Attention-Deficit/Hyperactivity Disorder
ALAT: Alanine aminotransferase
ANA: Antinuclear Antibody
Anti-TPO: Thyroid Peroxidase antibodies
APGAR: Appearance, Pulse, Grimace, Activity, Respiration
APP: Acute Phase Protein
AQ: Adult Autism Spectrum Quotient
ASR: Adult Self Report
ASRS: Attention-Deficit/Hyperactivity Disorder Self-Report Scale
ATC: Anatomical Therapeutic Chemical
BMI: Body-Mass Index
BOCS: Brief Obsessive-Compulsive Scale
BRAVO 3D: Axial MRI 3D Brain Volume
CAT-Q: Camouflaging Autistic Traits Questionnaire
CATSS: Child and Adolescent Twin Study in Sweden
CBC: Complete Blood Count
CNV: Copy Number Variation
CpG: Cytosine-phosphate-Guanine
CREAT: Comprehensive Risk Evaluation for Anorexia nervosa in Twins
CRP: C-Reactive Protein
DLGAP3: DLG Associated Protein 3
DMN: Default Mode Network
DNA: Deoxyribonucleic Acid
DOGSS: Developmental Outcomes in a Genetics twin Study in Sweden
DSM-5: Diagnostic and Statistical Manual of mental disorders, fifth edition
DTI: Diffusion Tensor Imaging
EPI: Echo-Planar Images
ESR: Erythrocyte Sedimentation Rate
FA: Fractional Anisotropy
FOV: Field Of View
FPT: Fragmented Picture Test
GEE: Generalized Estimating Equation
GO: Gene Ontology
GWAS: Genome-wide Association Study
HARDI: High AngulaR Diffusion Imaging
IL: Interleukin
K-SADS-PL: Kiddie-Schedule for Affective Disorders and Schizophrenia for school-aged children, present and lifetime Version
LCMS: Liquid Chromatography-Mass Spectrometry
MHz: Megahertz
MRI: Magnetic Resonance Imaging
MZ: MonoZygotic
NMR: Nuclear Magnetic Resonance
NRXN1: Neurexin 1
PANDAS: Pediatric Autoimmune Neuropsychiatric Disorder Associated with Streptococcal Infection
PCR: Polymerase Chain Reaction
OCD: Obsessive-Compulsive Disorder
OCI-R: Obsessive-Compulsive Inventory-Revised
PKU: PhenylKetonUria
RATSS: Roots of Autism and Attention-Deficit/Hyperactivity Disorder Twin Study in Sweden
rDNA: ribosomal Deoxyribonucleic Acid
RefSeq: Reference Sequence
rsfMRI: resting state functional Magnetic Resonance Imaging
rRNA: ribosomal Ribonucleic Acid
RTMITE: Reading the mind in the eyes
SCID-5: Structured Clinical Interview for DSM-5 Axis I disorders
SEK: Swedish Krona
SNP: Single Nucleotide Polymorphism
SP-A: Sensory Profile-Adolescent/Adult
STAGE: Screening Twin Adults: Genes and Environment study
SWEAA: Swedish Eating Assessment for Autism spectrum disorders
T4: Thyroxine
TNF-α: Tumor Necrosis Factor-α human
TR/TE: Repetition Time/Echo Time
TSH: Thyroid Stimulating Hormone
WAIS-IV: Wechsler Adult Intelligence Scale-IV
WGS: Whole Genome Sequencing
YATSS: Young Adult Twins in Sweden Study
Y-BOCS: Yale-Brown Obsessive-Compulsive Scale

## References

[1] Mataix-Cols D, Hansen B, Mattheisen M, Karlsson EK, Addington AM, Boberg J, Djurfeldt DR, Halvorsen M, Lichtenstein P, Solem S, Nordic OCD & Related Disorders Consortium: Rationale, design, and methods. Am J Med Genet B Neuropsychiatr Genet 2020, 183(1):38–50. 31424634 10.1002/ajmg.b.32756 PMC6898732

[2] Mahjani B, Dellenvall K, Grahnat AS, Karlsson G, Tuuliainen A, Reichert J, Mahjani CG, Klei L, De Rubeis S, Reichenberg A, Cohort profile: Epidemiology and Genetics of Obsessive-compulsive disorder and chronic tic disorders in Sweden (EGOS). Soc Psychiatry Psychiatr Epidemiol 2020, 55(10):1383–1393. 31907560 10.1007/s00127-019-01822-7

[3] NI Strom, Yu D, Gerring ZF, Halvorsen MW, Abdellaoui A, Rodriguez-Fontenla C, Sealock JM, Bigdeli T, Coleman JRI, Mahjani B, Genome-wide association study identifies new locus associated with OCD. medRxiv 2021:2021.2010.2013.21261078.

[4] Mataix-Cols D, Boman M, Monzani B, Ruck C, Serlachius E, Langstrom N, Lichtenstein P. Population-based, multigenerational family clustering study of obsessive-compulsive disorder. JAMA Psychiatry 2013, 70(7):709–717. 23699935 10.1001/jamapsychiatry.2013.3

[5] Brander G, Perez-Vigil A, Larsson H, Mataix-Cols D. Systematic review of environmental risk factors for Obsessive-Compulsive Disorder: A proposed roadmap from association to causation. Neurosci Biobehav Rev 2016, 65:36–62. 27013116 10.1016/j.neubiorev.2016.03.011

[6] Ludvigsson JF, Almqvist C, Bonamy AKE, Ljung R, Michaelsson K, Neovius M, Stephansson O, Ye WM. Registers of the Swedish total population and their use in medical research. Eur J Epidemiol 2016, 31(2):125–136. 26769609 10.1007/s10654-016-0117-y

[7] Magnusson PK, Almqvist C, Rahman I, Ganna A, Viktorin A, Walum H, Halldner L, Lundstrom S, Ullen F, Langstrom N, The Swedish Twin Registry: establishment of a biobank and other recent developments. Twin Res Hum Genet 2013, 16(1):317–329. 23137839 10.1017/thg.2012.104

[8] Zagai U, Lichtenstein P, Pedersen NL, Magnusson PKE. The Swedish Twin Registry: Content and Management as a Research Infrastructure. Twin Res Hum Genet 2019, 22(6):672–680. 31747977 10.1017/thg.2019.99

[9] PKU-biobanken [The PKU biobank]. Accessed from: https://www.karolinska.se/for-vardgivare/karolinska-universitetslaboratoriet/centrum-for-medfodda-metabola-sjukdomar/pku-biobank/

[10] Bölte S, Willfors C, Berggren S, Norberg J, Poltrago L, Mevel K, Coco C, Fransson P, Borg J, Sitnikov R, The Roots of Autism and ADHD Twin Study in Sweden (RATSS). Twin Res Hum Genet 2014, 17(3):164–176. 24735654 10.1017/thg.2014.12

[11] Lichtenstein P, Sullivan PF, Cnattingius S, Gatz M, Johansson S, Carlstrom E, Bjork C, Svartengren M, Wolk A, Klareskog L, The Swedish Twin Registry in the third millennium: an update. Twin Res Hum Genet 2006, 9(6):875–882. 17254424 10.1375/183242706779462444

[12] Ludvigsson JF, Andersson E, Ekbom A, Feychting M, Kim JL, Reuterwall C, Heurgren M, Olausson PO. External review and validation of the Swedish national inpatient register. BMC Public Health 2011, 11:450. 21658213 10.1186/1471-2458-11-450 PMC3142234

[13] Stamouli S, Anderlid BM, Willfors C, Thiruvahindrapuram B, Wei J, Berggren S, Nordgren A, Scherer SW, Lichtenstein P, Tammimies K, Copy Number Variation Analysis of 100 Twin Pairs Enriched for Neurodevelopmental Disorders. Twin Res Hum Genet 2018, 21(1):1–11. 29307321 10.1017/thg.2017.69

[14] Goetgeluk S, Vansteelandt S. Conditional generalized estimating equations for the analysis of clustered and longitudinal data. Biometrics 2008, 64(3):772–780. 18047524 10.1111/j.1541-0420.2007.00944.x

[15] Neuhaus JM, McCulloch CE. Separating between- and within-cluster covariate effects by using conditional and partitioning methods. J Roy Stat Soc B 2006, 68:859–872.

[16] Allison P. Fixed Effects Regression Models, Quantitative Applications in the Social Sciences Vol. 160. Thousand Oaks, California: SAGE; 2009.

[17] Sjolander A, Zetterqvist J. Confounders, Mediators, or Colliders What Types of Shared Covariates Does a Sibling Comparison Design Control For? Epidemiology 2017, 28(4):540–547. 28575894 10.1097/EDE.0000000000000649

[18] Ludvigsson JF, Otterblad-Olausson P, Pettersson BU, Ekbom A. The Swedish personal identity number: possibilities and pitfalls in healthcare and medical research. Eur J Epidemiol 2009, 24(11):659–667. 19504049 10.1007/s10654-009-9350-y PMC2773709

[19] Anckarsater H, Lundstrom S, Kollberg L, Kerekes N, Palm C, Carlstrom E, Langstrom N, Magnusson PKE, Halldner L, Bolte S, The Child and Adolescent Twin Study in Sweden (CATSS). Twin Res Hum Genet 2011, 14(6):495–508. 22506305 10.1375/twin.14.6.495

[20] Pauls DL, Abramovitch A, Rauch SL, Geller DA. Obsessive-compulsive disorder: an integrative genetic and neurobiological perspective. Nat Rev Neurosci 2014, 15(6):410–424. 24840803 10.1038/nrn3746

[21] Barbosa M, Joshi RS, Garg P, Martin-Trujillo A, Patel N, Jadhav B, Watson CT, Gibson W, Chetnik K, Tessereau C, Identification of rare de novo epigenetic variations in congenital disorders. Nat Commun 2018, 9(1):2064. 29802345 10.1038/s41467-018-04540-x PMC5970273

[22] Nissen JB, Hansen CS, Starnawska A, Mattheisen M, Borglum AD, Buttenschon HN, Hollegaard M. DNA Methylation at the Neonatal State and at the Time of Diagnosis: Preliminary Support for an Association with the Estrogen Receptor 1, Gamma-Aminobutyric Acid B Receptor 1, and Myelin Oligodendrocyte Glycoprotein in Female Adolescent Patients with OCD. Front Psychiatry 2016, 7:35. 27047397 10.3389/fpsyt.2016.00035 PMC4796012

[23] Mattheisen M, Samuels JF, Wang Y, Greenberg BD, Fyer AJ, McCracken JT, Geller DA, Murphy DL, Knowles JA, Grados MA, Genome-wide association study in obsessive-compulsive disorder: results from the OCGAS. Mol Psychiatry 2015, 20(3):337–344. 24821223 10.1038/mp.2014.43 PMC4231023

[24] Stewart SE, Yu D, Scharf JM, Neale BM, Fagerness JA, Mathews CA, Arnold PD, Evans PD, Gamazon ER, Osiecki L, Genome-wide association study of obsessive-compulsive disorder. Mol Psychiatry 2013, 18(7):788–798. 22889921 10.1038/mp.2012.85 PMC4218751

[25] Arnold PD, Askland KD, Barlassina C, Bellodi L, Bienvenu OJ, Black D, Bloch M, Brentani H, Burton CL, Camarena B, Revealing the complex genetic architecture of obsessive-compulsive disorder using meta-analysis. Mol Psychiatry 2018, 23(5):1181–1188. 28761083 10.1038/mp.2017.154 PMC6660151

[26] Rahmani E, Schweiger R, Rhead B, Criswell LA, Barcellos LF, Eskin E, Rosset S, Sankararaman S, Halperin E. Cell-type-specific resolution epigenetics without the need for cell sorting or single-cell biology. Nat Commun 2019, 10(1):3417. 31366909 10.1038/s41467-019-11052-9 PMC6668473

[27] Matias A, Silva S, Martins Y, Blickstein I. Monozygotic twins: Ten reasons to be different. Diagnóstico Prenatal 2014, 25(2):53–57.

[28] Jonsson H, Magnusdottir E, Eggertsson HP, Stefansson OA, Arnadottir GA, Eiriksson O, Zink F, Helgason EA, Jonsdottir I, Gylfason A, Differences between germline genomes of monozygotic twins. Nat Genet 2021, 53(1):27–34. 33414551 10.1038/s41588-020-00755-1

[29] Gardner RM, Dalman C, Wicks S, Lee BK, Karlsson H. Neonatal levels of acute phase proteins and later risk of non-affective psychosis. Transl Psychiatry 2013, 3:e228. 23423137 10.1038/tp.2013.5 PMC3591005

[30] Blomstrom A, Gardner RM, Dalman C, Yolken RH, Karlsson H. Influence of maternal infections on neonatal acute phase proteins and their interaction in the development of non-affective psychosis. Transl Psychiatry 2015, 5:e502. 25646591 10.1038/tp.2014.142 PMC4445745

[31] Dahlin AM, Hollegaard MV, Wibom C, Andersson U, Hougaard DM, Deltour I, Hjalmars U, Melin B. CCND2, CTNNB1, DDX3X, GLI2, SMARCA4, MYC, MYCN, PTCH1, TP53, and MLL2 gene variants and risk of childhood medulloblastoma. J Neurooncol 2015, 125(1):75–78. 26290144 10.1007/s11060-015-1891-1 PMC4592490

[32] Swedo SE. Pediatric autoimmune neuropsychiatric disorders associated with streptococcal infections (PANDAS). Mol Psychiatry 2002, 7 Suppl 2:S24–25. 12142939 10.1038/sj.mp.4001170

[33] Zhang T, Brander G, Isung J, Isomura K, Sidorchuk A, Larsson H, Chang Z, Mataix-Cols D, Fernandez de la Cruz L. Prenatal and Early Childhood Infections and Subsequent Risk of Obsessive-Compulsive Disorder and Tic Disorders: A Nationwide, Sibling-Controlled Study. Biol Psychiatry 2022. 10.1016/j.biopsych.2022.07.004 36155699

[34] Turna J, Grosman Kaplan K, Anglin R, Van Ameringen M. "What's Bugging the Gut in Ocd?" a Review of the Gut Microbiome in Obsessive-Compulsive Disorder. Depress Anxiety 2016, 33(3):171–178. 26629974 10.1002/da.22454

[35] Cryan JF, Dinan TG. Mind-altering microorganisms: the impact of the gut microbiota on brain and behaviour. Nat Rev Neurosci 2012, 13(10):701–712. 22968153 10.1038/nrn3346

[36] Rajendhran J, Gunasekaran P. Microbial phylogeny and diversity: Small subunit ribosomal RNA sequence analysis and beyond. Microbiol Res 2011, 166(2):99–110. 20223646 10.1016/j.micres.2010.02.003

[37] Menzies L, Chamberlain SR, Laird AR, Thelen SM, Sahakian BJ, Bullmore ET. Integrating evidence from neuroimaging and neuropsychological studies of obsessive-compulsive disorder: the orbitofronto-striatal model revisited. Neurosci Biobehav Rev 2008, 32(3):525–549. 18061263 10.1016/j.neubiorev.2007.09.005 PMC2889493

[38] Chamberlain SR, Menzies L, Hampshire A, Suckling J, Fineberg NA, del Campo N, Aitken M, Craig K, Owen AM, Bullmore ET, Orbitofrontal dysfunction in patients with obsessive-compulsive disorder and their unaffected relatives. Science 2008, 321(5887):421–422. 18635808 10.1126/science.1154433

[39] de Wit SJ, Froukje E., de Vries MD, van der Werf YD, Cath DC, Heslenfeld DJ, Veltman EM, van Balkom AJLM, Presupplementary Motor Area Hyperactivity During Response Inhibition: A Candidate Endophenotype of Obsessive-Compulsive Disorder. Am J Psychiat 2012, 169(10):1100–1108. 23032388 10.1176/appi.ajp.2012.12010073

[40] Hou JM, Zhao M, Zhang W, Song LH, Wu WJ, Wang J, Zhou DQ, Xie B, He M, Guo JW, Resting-state functional connectivity abnormalities in patients with obsessive-compulsive disorder and their healthy first-degree relatives. J Psychiatry Neurosci 2014, 39(5):304–311. 24866415 10.1503/jpn.130220 PMC4160359

[41] Boedhoe PSW, Schmaal L, Abe Y, Alonso P, Ameis SH, Anticevic A, Arnold PD, Batistuzzo MC, Benedetti F, Beucke JC, Cortical Abnormalities Associated With Pediatric and Adult Obsessive-Compulsive Disorder: Findings From the ENIGMA Obsessive-Compulsive Disorder Working Group. Am J Psychiat 2018, 175(5):453–462. 29377733 10.1176/appi.ajp.2017.17050485 PMC7106947

[42] Boedhoe PSW, Schmaal L, Abe Y, Ameis SH, Arnold PD, Batistuzzo MC, Benedetti F, Beucke JC, Bollettini I, Bose A, Distinct Subcortical Volume Alterations in Pediatric and Adult OCD: A Worldwide Meta-and Mega-Analysis. Am J Psychiat 2017, 174(1):60–69. 27609241 10.1176/appi.ajp.2016.16020201 PMC5344782

[43] Fouche JP, du Plessis S, Hattingh C, Roos A, Lochner C, Soriano-Mas C, Sato JR, Nakamae T, Nishida S, Kwon JS, Cortical thickness in obsessive compulsive disorder: multisite mega-analysis of 780 brain scans from six centres. Br J Psychiatry 2017, 210(1):67–74. 27198485 10.1192/bjp.bp.115.164020

[44] de Wit SJ, Alonso P, Schweren L, Mataix-Cols D, Lochner C, Menchon JM, Stein DJ, Fouche JP, Soriano-Mas C, Sato JR, Multicenter voxel-based morphometry mega-analysis of structural brain scans in obsessive-compulsive disorder. Am J Psychiatry 2014, 171(3):340–349. 24220667 10.1176/appi.ajp.2013.13040574

[45] Bruin WB, Taylor L, Thomas RM, Shock JP, Zhutovsky P, Abe Y, Alonso P, Ameis SH, Anticevic A, Arnold PD, Structural neuroimaging biomarkers for obsessive-compulsive disorder in the ENIGMA-OCD consortium: medication matters. Transl Psychiatry 2020, 10(1):342. 33033241 10.1038/s41398-020-01013-y PMC7598942

[46] Ivanov I, Boedhoe PSW, Abe Y, Alonso P, Ameis SH, Arnold PD, Balachander S, Baker JT, Banaj N, Bargallo N, Associations of medication with subcortical morphology across the lifespan in OCD: Results from the international ENIGMA Consortium. J Affect Disord 2022, 318:204–216. 36041582 10.1016/j.jad.2022.08.084

[47] Radua J, van den Heuvel OA, Surguladze S, Mataix-Cols D. Meta-analytical comparison of voxel-based morphometry studies in obsessive-compulsive disorder vs other anxiety disorders. Arch Gen Psychiatry 2010, 67(7):701–711. 20603451 10.1001/archgenpsychiatry.2010.70

[48] Radua J, Grau M, van den Heuvel OA, Thiebaut de Schotten M, Stein DJ, Canales-Rodriguez EJ, Catani M, Mataix-Cols D. Multimodal voxel-based meta-analysis of white matter abnormalities in obsessive-compulsive disorder. Neuropsychopharmacology 2014, 39(7):1547–1557. 24407265 10.1038/npp.2014.5 PMC4023155

[49] Piras F, Piras F, Abe Y, Agarwal SM, Anticevic A, Ameis S, Arnold P, Banaj N, Bargallo N, Batistuzzo MC, White matter microstructure and its relation to clinical features of obsessive-compulsive disorder: findings from the ENIGMA OCD Working Group. Transl Psychiatry 2021, 11(1):173. 33731673 10.1038/s41398-021-01276-z PMC7969744

[50] Beucke JC, Sepulcre J, Talukdar T, Linnman C, Zschenderlein K, Endrass T, Kaufmann C, Kathmann N. Abnormally high degree connectivity of the orbitofrontal cortex in obsessive-compulsive disorder. JAMA Psychiatry 2013, 70(6):619–629. 23740050 10.1001/jamapsychiatry.2013.173

[51] Harrison BJ, Soriano-Mas C, Pujol J, Ortiz H, Lopez-Sola M, Hernandez-Ribas R, Deus J, Alonso P, Yucel M, Pantelis C, Altered corticostriatal functional connectivity in obsessive-compulsive disorder. Arch Gen Psychiatry 2009, 66(11):1189–1200. 19884607 10.1001/archgenpsychiatry.2009.152

[52] Figee M, Luigjes J, Smolders R, Valencia-Alfonso CE, van Wingen G, de Kwaasteniet B, Mantione M, Ooms P, de Koning P, Vulink N, Deep brain stimulation restores frontostriatal network activity in obsessive-compulsive disorder. Nat Neurosci 2013, 16(4):386–387. 23434914 10.1038/nn.3344

[53] Beucke JC, Sepulcre J, Eldaief MC, Sebold M, Kathmann N, Kaufmann C. Default mode network subsystem alterations in obsessive-compulsive disorder. Br J Psychiatry 2014, 205(5):376–382. 25257066 10.1192/bjp.bp.113.137380

[54] Hawrylycz MJ, Lein ES, Guillozet-Bongaarts AL, Shen EH, Ng L, Miller JA, van de Lagemaat LN, Smith KA, Ebbert A, Riley ZL, An anatomically comprehensive atlas of the adult human brain transcriptome. Nature 2012, 489(7416):391–399. 22996553 10.1038/nature11405 PMC4243026

[55] Diez I, Sepulcre J. Neurogenetic profiles delineate large-scale connectivity dynamics of the human brain. Nat Commun 2018, 9(1):3876. 30250030 10.1038/s41467-018-06346-3 PMC6155203

[56] Ortiz-Teran L, Diez I, Ortiz T, Perez DL, Aragon JI, Costumero V, Pascual-Leone A, El Fakhri G, Sepulcre J. Brain circuit-gene expression relationships and neuroplasticity of multisensory cortices in blind children. Proc Natl Acad Sci U S A 2017, 114(26):6830–6835. 28607055 10.1073/pnas.1619121114 PMC5495230

[57] Sepulcre J, Grothe MJ, d'Oleire Uquillas F, Ortiz-Teran L, Diez I, Yang HS, Jacobs HIL, Hanseeuw BJ, Li Q, El-Fakhri G, Neurogenetic contributions to amyloid beta and tau spreading in the human cortex. Nat Med 2018, 24(12):1910–1918. 30374196 10.1038/s41591-018-0206-4 PMC6518398

[58] Zuchner S, Wendland JR, Ashley-Koch AE, Collins AL, Tran-Viet KN, Quinn K, Timpano KC, Cuccaro ML, Pericak-Vance MA, Steffens DC, Multiple rare SAPAP3 missense variants in trichotillomania and OCD. Mol Psychiatry 2009, 14(1):6–9. 19096451 10.1038/mp.2008.83 PMC2803344

[59] Bienvenu OJ, Wang Y, Shugart YY, Welch JM, Grados MA, Fyer AJ, Rauch SL, McCracken JT, Rasmussen SA, Murphy DL, Sapap3 and pathological grooming in humans: Results from the OCD collaborative genetics study. Am J Med Genet B Neuropsychiatr Genet 2009, 150B(5):710–720. 19051237 10.1002/ajmg.b.30897 PMC10885776

[60] Boardman L, van der Merwe L, Lochner C, Kinnear CJ, Seedat S, Stein DJ, Moolman-Smook JC, Hemmings SMJ. Investigating SAPAP3 variants in the etiology of obsessive-compulsive disorder and trichotillomania in the South African white population. Compr Psychiatry 2011, 52(2):181–187. 21295225 10.1016/j.comppsych.2010.05.007

[61] Welch JM, Lu J, Rodriguiz RM, Trotta NC, Peca J, Ding JD, Feliciano C, Chen M, Adams JP, Luo JH, Cortico-striatal synaptic defects and OCD-like behaviours in Sapap3-mutant mice. Nature 2007, 448(7156):894–U892. 17713528 10.1038/nature06104 PMC2442572

[62] Noh HJ, Tang RQ, Flannick J, O'Dushlaine C, Swofford R, Howrigan D, Genereux DP, Johnson J, van Grootheest G, Grunblatt E, Integrating evolutionary and regulatory information with multispecies approach implicates genes and pathways in obsessive-compulsive disorder. Nat Commun 2017, 8(1):774. 29042551 10.1038/s41467-017-00831-x PMC5645406

[63] Arora M, Reichenberg A, Willfors C, Austin C, Gennings C, Berggren S, Lichtenstein P, Anckarsater H, Tammimies K, Bolte S. Fetal and postnatal metal dysregulation in autism. Nat Commun 2017, 8:15493. 28569757 10.1038/ncomms15493 PMC5461492

[64] Neufeld J, Kuja-Halkola R, Mevel K, Cauvet E, Fransson P, Bolte S. Alterations in resting state connectivity along the autism trait continuum: a twin study. Mol Psychiatry 2018, 23(7):1659–1665. 28761079 10.1038/mp.2017.160

[65] Fernández de la Cruz L, Isomura K, Lichtenstein P, Ruck C, Mataix-Cols D. Morbidity and mortality in obsessive-compulsive disorder: A narrative review. Neurosci Biobehav Rev 2022, 136:104602. 35271916 10.1016/j.neubiorev.2022.104602

[66] Pérez-Vigil A, Fernández de la Cruz L, Brander G, Isomura K, Jangmo A, Feldman I, Hesselmark E, Serlachius E, Lazaro L, Ruck C, Association of Obsessive-Compulsive Disorder With Objective Indicators of Educational Attainment: A Nationwide Register-Based Sibling Control Study. JAMA Psychiatry 2018, 75(1):47–55. 29141084 10.1001/jamapsychiatry.2017.3523 PMC5833536

[67] Pérez-Vigil A, Mittendorfer-Rutz E, Helgesson M, Fernández de la Cruz L, Mataix-Cols D. Labour market marginalisation in obsessive-compulsive disorder: a nationwide register-based sibling control study. Psychol Med 2018:1–10. 10.1017/S0033291718001691 29950186

[68] Davis JO, Phelps JA, Bracha HS. Prenatal development of monozygotic twins and concordance for schizophrenia. Schizophr Bull 1995, 21(3):357–366. 7481567 10.1093/schbul/21.3.357

[69] Seidel M, Ehrlich S, Breithaupt L, Welch E, Wiklund C, Hubei C, Thornton LM, Savva A, Fundin BT, Pege J, Study protocol of comprehensive risk evaluation for anorexia nervosa in twins (CREAT): a study of discordant monozygotic twins with anorexia nervosa. BMC Psychiatry 2020, 20(1):507. 33054774 10.1186/s12888-020-02903-7 PMC7557028

[70] Axelsson O. The Swedish Medical Birth Register. Acta Obstet Gyn Scan 2003, 82(6):491–492. 10.1034/j.1600-0412.2003.00172.x 12780418

[71] Wettermark B, Hammar N, Fored CM, Leimanis A, Otterblad Olausson P, Bergman U, Persson I, Sundstrom A, Westerholm B, Rosen M. The new Swedish Prescribed Drug Register--opportunities for pharmacoepidemiological research and experience from the first six months. Pharmacoepidemiol Drug Saf 2007, 16(7):726–735. 16897791 10.1002/pds.1294

